# Increased nuclear DNA damage precedes mitochondrial dysfunction in peripheral blood mononuclear cells from Huntington’s disease patients

**DOI:** 10.1038/s41598-018-27985-y

**Published:** 2018-06-29

**Authors:** Georgina Askeland, Zaneta Dosoudilova, Marie Rodinova, Jiri Klempir, Irena Liskova, Anna Kuśnierczyk, Magnar Bjørås, Gaute Nesse, Arne Klungland, Hana Hansikova, Lars Eide

**Affiliations:** 1 Department of Medical Biochemistry, Institute of Clinical Medicine, University of Oslo, Oslo, Norway; 2 Department of Microbiology, Oslo University Hospital, Oslo, Norway; 3 Department of Pediatrics and Adolescent Medicine, Charles University in Prague and General University Hospital in Prague, Prague, Czech Republic; 4 Department of Neurology and Centre of Clinical Neuroscience, First Faculty of Medicine, Charles University in Prague and General University Hospital in Prague, Prague, Czech Republic; 5 Proteomics and Metabolomics Core Facility, PROMEC, Department of Clinical and Molecular Medicine, Norwegian University of Science and Technology, Trondheim, Norway

## Abstract

Huntington’s disease (HD) is a progressive neurodegenerative disorder primarily affecting the basal ganglia and is caused by expanded CAG repeats in the huntingtin gene. Except for CAG sizing, mitochondrial and nuclear DNA (mtDNA and nDNA) parameters have not yet proven to be representative biomarkers for disease and future therapy. Here, we identified a general suppression of genes associated with aerobic metabolism in peripheral blood mononuclear cells (PBMCs) from HD patients compared to controls. In HD, the complex II subunit SDHB was lowered although not sufficiently to affect complex II activity. Nevertheless, we found decreased level of factors associated with mitochondrial biogenesis and an associated dampening of the mitochondrial DNA damage frequency in HD, implying an early defect in mitochondrial activity. In contrast to mtDNA, nDNA from HD patients was four-fold more modified than controls and demonstrated that nDNA integrity is severely reduced in HD. Interestingly, the level of nDNA damage correlated inversely with the total functional capacity (TFC) score; an established functional score of HD. Our data show that PBMCs are a promising source to monitor HD progression and highlights nDNA damage and diverging mitochondrial and nuclear genome responses representing early cellular impairments in HD.

## Introduction

Huntington’s disease is an autosomal dominant progressive neurodegenerative disorder caused by an inherited, pathogenic expansion of CAG repeats in exon 1 of the huntingtin gene (*HTT*). The mutant HTT (mHTT) protein manifests into HD when the CAG tracts expands beyond 36; while wild type HTT contains between 16 and 25 CAG repeats^1^. The size of the expansion is correlated with disease severity, with increasing CAG repeat lengths accelerating the age of onset. The HTT gene encodes a 350 kDa protein whose exact function is still not understood. The gene is essential for viability and is ubiquitously expressed^2–4^. The mHTT protein influences a plethora of cellular processes including transcription, axonal transport, cytoskeletal structure/function, signal transduction, and autophagy that ultimately result in cell death. HD predominantly affects the basal ganglia, leading to affective, cognitive, behavioural and motor decline, which then progresses to death. Medium GABAergic spiny neurons in the striatal part of the basal ganglia are particularly vulnerable. HD patients exhibit heterogeneous disease progression. The Unified Huntington’s Disease Rating Score (UHDRS) was established as a reliable hybrid scale to describe motor function, cognitive function, behaviour abnormalities, and total functional capacity (TFC)^5^, that together with molecular genetic testing (CAG sizing)^6^ provided tools to monitor disease. It has nevertheless been reported that loss of caudate volume exceeds 50% by the time of diagnosis^7^, and premanifest *mHTT* carriers display striatal atrophy 15–20 years before predicted disease onset^8^ The heterogeneity of the disease is further illustrated by that tests for behaviour, motor score, functional and cognitive decline do not overlap^9^.

Although neurological symptoms have been the main focus of the disease, mHTT exerts pathogenic effects in other tissues as well. Predisposition to diabetes and skeletal muscle wasting that precedes detectable neurodegeneration has been reported in patients, demonstrating that HD is a systemic disease^10,11^. Mouse models of HD demonstrate impaired glucose uptake and altered gene regulation^12^, as well as cardiac dysfunction^13^.

Mitochondrial alterations are well documented in HD. Immortalized lymphoblasts from HD patients are hypersensitive to mitochondrial inhibition^14^, and homozygous *mHTT* cells display mitochondrial aberrations^15^. Primary HD blood cells are characterized by increased levels of pro-apoptotic proteins and reactive oxygen species (ROS)^16^. The HD cellular phenotype is reminiscent of mitochondrial dysfunction. This association is emphasized by the HD-mimicking striatal degeneration by the mycotoxin 3-nitropropionic acid; a mitochondrial complex II inhibitor that was accidentally consumed as contamination of sugarcanes^17^. Subsequently, systemic exposure to 3-nitropropionic acid was introduced to investigate striatal neurodegeneration in rodents and verify the coupling between mitochondrial electron transport chain (ETC) complex II dysfunction and striatal degeneration.

Mitochondrial dysfunction is a general feature of mouse models of HD. The *mHtt* knock-in model carries an expanded CAG tract (150 CAGs) in the endogenous murine gene and represents a physiological relevant model to understand the initial phases of the disease^18^. The mHTT protein in this model influences nuclear gene expression including PGC-1α, the major regulator of mitochondrial biogenesis and result in reduced thermoregulation and reduced expression of the dismutases SOD1 and SOD2^19^. Polyglutamines interfere with mitochondria and disturb intracellular trafficking and cause mitochondrial fragmentation^20,21^.

The extended CAG tract in the *mHTT* gene is genetically unstable, and increases with age in a tissue-specific manner, as shown in both patients and HD models. Studies from mouse models indicate that faulty DNA repair underlies somatic expansions in the polyQ tract, in particular the DNA base excision repair factors OGG1 and, NEIL1^22,23^ and the DNA mismatch repair protein MSH2^24^.

These observations imply that mitochondrial aberrations and/or DNA maintenance are key parameters underlying the disease. The abrogation of somatic expansions in MSH2 deficiency delayed nuclear mHTT aggregation and such implied that somatic expansion drives the progression of the disease^25^^,^^26^.

We hypothesized that mHTT would systemically affect mitochondrial function and that peripheral blood mononuclear cells (PBMCs) as post-mitotic (and peripheral) cells would serve as suitable and easy accessible material presenting HD. Parameters of oxidative metabolism, including mitochondrial ETC assessment were assessed to investigate cellular energy status. DNA damage was included as a quantitative measure of the intracellular redox state.

## Results

### Early mitochondrial aberrations in HD PBMCs

**Figure 1:**
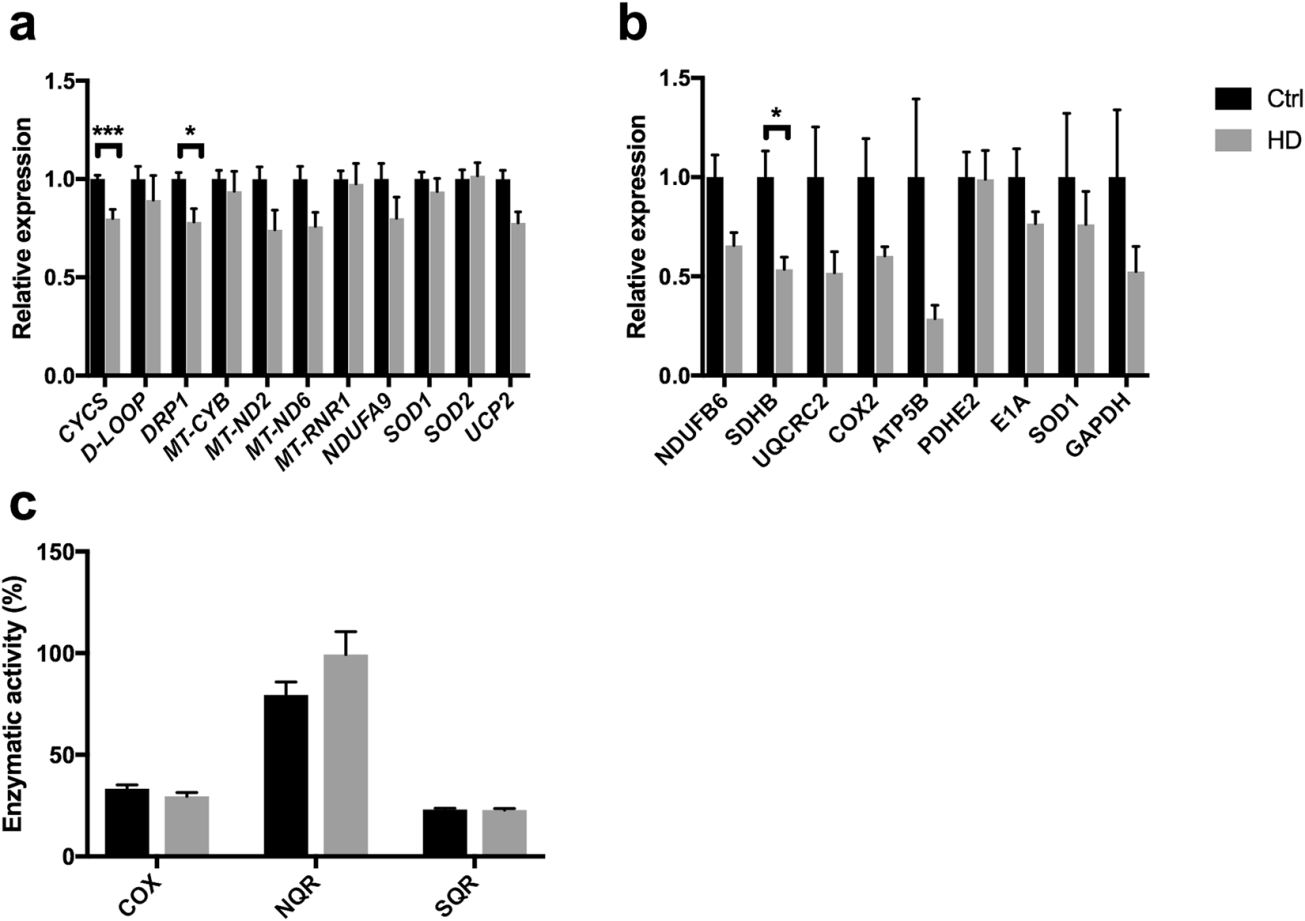
Early mitochondrial alterations in HD PBMCs. (**a**) Expression of mitochondria-related genes in HD (n = 12–18) relative to controls (n = 32–44). (**b**) Expression of proteins associated with mitochondrial function in HD (n = 17) and controls (n = 5). (**c**) Biochemical ETC complex activity in HD (n = 17) and control (n = 34) PBMCs. Data represent mean with error bars representing standard error mean. *P < 0.05., ***P < 0.001.

PBMCs were isolated from 36 patients and 49 healthy volunteers (controls). The patients were genetically tested for the CAG size. The sampling of PBMCs was paralleled by an assessment of the TFC, which was performed as part of the clinical follow-up. An overview of the included cohort is provided in Table 1. TFC was inversely correlated with duration of disease (R = −0.77, P < 0.001). There is partial correlation between onset of disease and TFC when age is corrected for (R = 0.61, P < 0.05).To investigate the influence of HD on mitochondrial function, we assessed the expression of mtDNA- and nDNA encoded genes related to mitochondrial pathways. We found significant downregulation of genes related to mitochondrial function in HD PBMCs. These are *CYCS and DRP1* (P < 0.05) and *UCP2* at the borderline after adjustment for multiple comparisons (Fig. 1a). The mtDNA-encoded genes *MT-ND2, MT-ND6* genes and mitochondrial small ribosomal subunit (*MT-RNR1*) were unaffected. RNA prepared from 18 individual HD PBMCs were grouped into three categories according to CAG repeat length (low-CAG, mid-CAG and high-CAG), pooled in equimolar ratios in each category, and subjected to RNA sequencing analyses together with pooled RNA in equimolar ratios from 18 controls. The RNA sequencing analyses confirmed the suppression of genes related to mitochondrial regulation, aerobic metabolism and oxidative stress (Supplementary Table S2). We did not observe a significant reduced level of mitochondria-associated proteins in HD (Fig. 1b). However, the reduction of the iron-sulfur containing subunit B of succinate dehydrogenase (SDHB) reached statistical significance after correction for multiple testing. Importantly, the reduction in SDHB did not correlate with CAG, age or TFC. When enzymatic activities of the ETC complexes were assessed, it became clear that the SDHB reduction did not produce a net effect on complex II (SQR). Likewise, the average complex I (NQR) and complex IV (COX) activities were not different in HD compared to controls (Fig. 1c). The ETC complexes altogether contain up to 100 different subunits, and although only a minor proportion of these were assessed in Fig. 1b, the present results imply at least that other subunits that could be affected in HD, are not bottlenecks for the ETC mitochondrial biochemical complex activity.

**Table 1:** Overview of included HD patients and age-matched controls.

		HD			Controls		
		Median	Range	(n)	Median	Range	(n)
Age (years)	All	41.5	[22–74]	36	46	[21–76]	49
	Men	40	[31–73]	19	41	[21–76]	25
	Women	46	[22–74]	17	47	[24–66]	24
CAG repeat size	All	45	[40–58]	36	N/A		
	Men	45	[41–53]	19	N/A		
	Women	44	[40–58]	17	N/A		
TFC score	All	7	[0–13]	36	N/A		
	Men	8	[1–13]	19	N/A		
	Women	6	[0–11]	17	N/A

**Figure 2:**
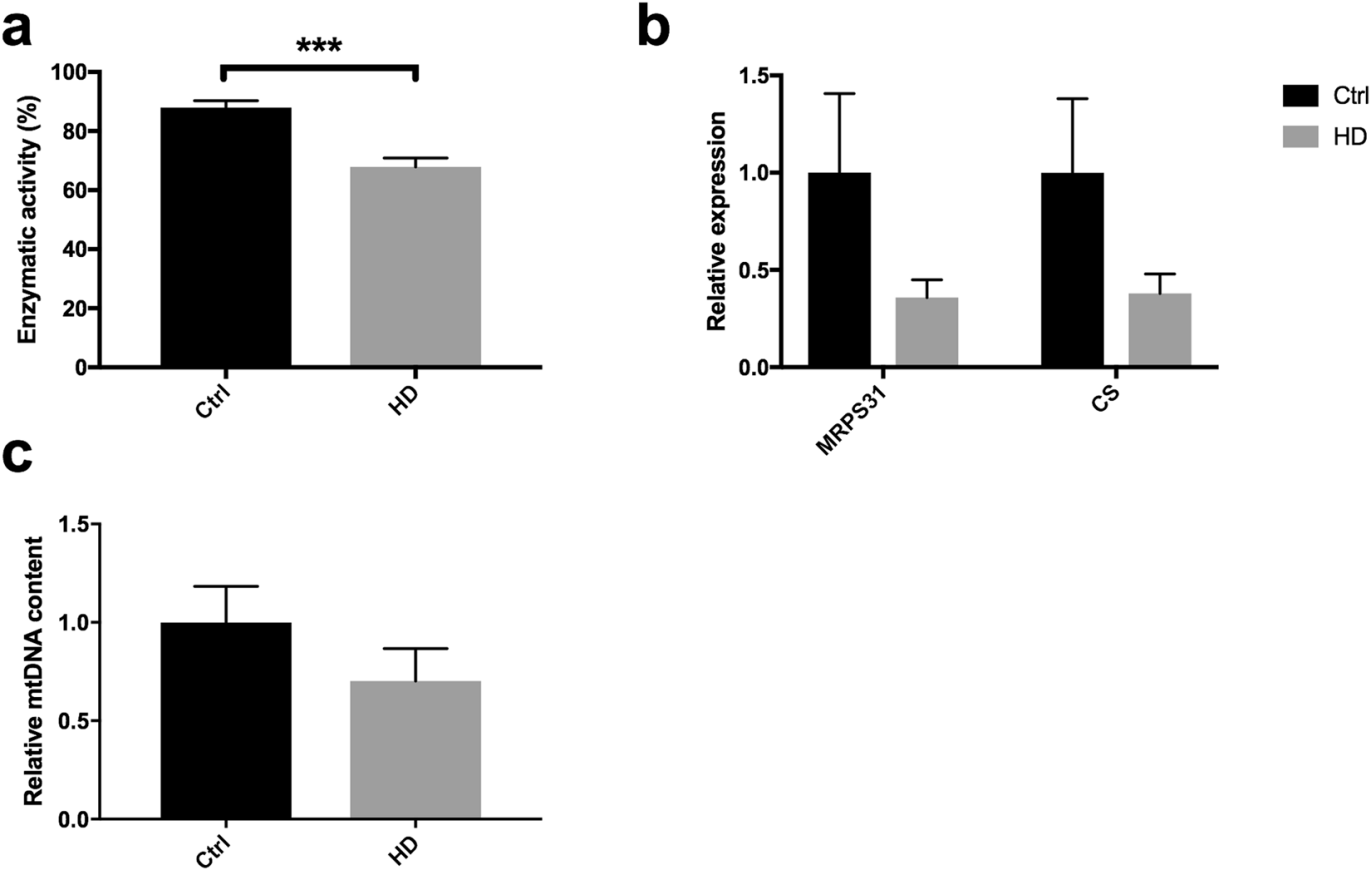
Mitochondrial biomarkers indicate reduced mitochondrial activity in HD. (**a**) Activity of citrate synthase in HD PBMCs (n = 17) relative to controls (n = 32). (**b**) Levels of ribosomal subunit MRPS31 and citrate synthase in HD (n = 17) and control (n = 5) PBMCs. (**c**) mtDNA copy number in HD and control PBMCs. ***P < 0.001.

Despite ETC being unaffected in HD, the level and activity of specific mitochondrial proteins were assessed more in detail, as cellular ETC complex capacity by itself does not necessarily reflect aerobic activity^27^. The activity of citrate synthase, a commonly used marker for mitochondrial content, was indeed significantly reduced in HD (−25%; Fig. 2a). The corresponding levels of the citrate synthase protein was reduced as well, similarly as the small mitochondrial ribosomal subunit MRPS31, both with borderline significance (P = 0.05) (Fig. 2b). The reduction in the above parameters was however not coherent with the stable expression of *MT-RNR1* (Fig. 1a) and might be indicative of an imbalanced composition of mitochondria in HD PBMCs. Interestingly, while there was a perfect symmetry between the activity of citrate synthase, complex I, II and IV in controls, the complex I activity did not correlate with any of the other activities in HD (Supplementary Table S2). The mtDNA copy number varies upon bioenergetic stimuli and oxidative stress. Upon its quantification, we found no difference between HD and controls (Fig. 2c).

### Genome integrity is affected in HD

**Figure 3:**
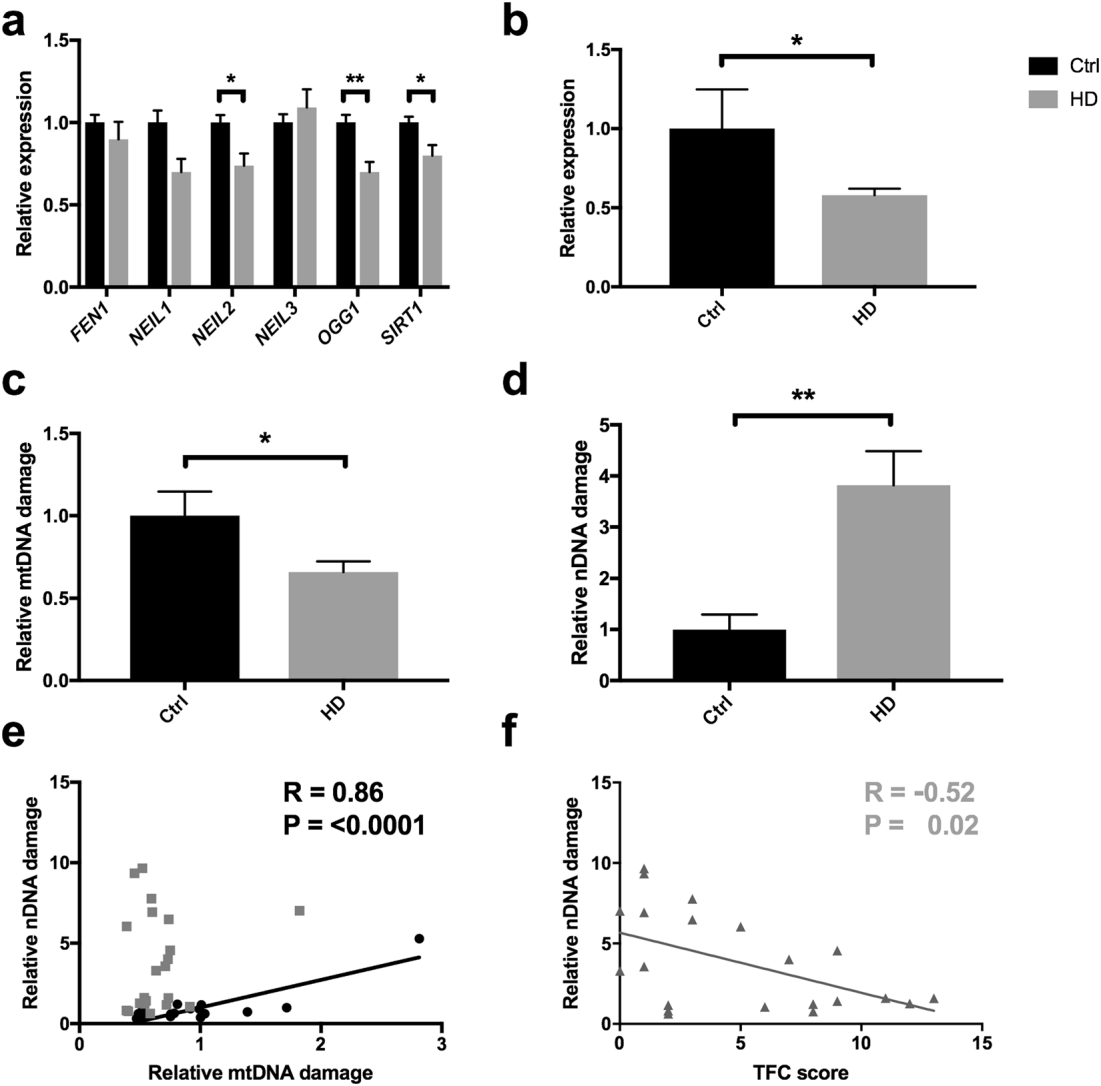
DNA integrity is impaired in HD PBMCs. (**a**) Expression of genes associated to base excision repair, in HD (n = 18) relative to controls (n = 44). (**b**) Expression of p53 protein level in HD (n = 17) and controls (n = 5). (**c**) mtDNA integrity and (**d**) nDNA integrity in HD (n = 21) and control (n = 16) PBMCs. (**e**) Correlation analyses between nDNA damage and mtDNA damage in HD and control PBMCs (n = 16). (**f**) Correlation analyses between nuclear DNA damage in PBMCs and TFC score in HD patients (n = 21) *P < 0.05; **P < 0.01. Pearson correlation with 95% confidence interval.

The overall mitochondrial aberration in HD PBMCs suggested that patient cells undergo metabolic alteration. We recently demonstrated that mtDNA responds to alterations in mitochondrial metabolism and that DNA repair factors influence the modification of mtDNA upon metabolic manipulations^28^. We therefore assessed the expression of genes associated to the base excision repair pathway, and found a tendency for general suppression of DNA repair capacity in HD (Fig. 3a). *OGG1* was most significantly suppressed. We followed up by assessing the protein level of p53, whose role is implicated in DNA repair as well as mitochondrial function. In contrast to earlier studies^29^, we found p53 level to be reduced in HD PBMCs (Fig. 3b), which supported the lowering of mitochondrial parameters (Fig. 2). To assess DNA integrity, we used a qPCR-based method^30^, which detects any modification that inhibits the TaqαI restriction enzyme. In line with the tendency to lower mitochondrial activity, the mtDNA damage in HD PBMCs was reduced by one third compared to controls (Fig. 3c). The lower mtDNA damage level was not likely to be due to excessive/mitophagic removal of damaged molecules, as the copy number of mtDNA was similar in both genotypes (Fig. 2c).

In contrast, a 4-fold (P < 0.01) increased level of nDNA damage was identified in HD PBMCs compared to healthy controls (Fig. 3d). We followed up by investigating the genomic DNA by mass spectrometry analyses. Despite the 4-fold increased nDNA damage level as measured by the qPCR-based method, we could not find any significant increase in any of the investigated base lesions/epigenetic marks in HD (Supplementary Fig. S1). Therefore, genomic alterations other than base lesions are likely causes of the elevated nDNA damage in HD.

These results suggest that mtDNA is relatively spared in HD compared to nDNA, and thus damaging events in the two cellular genomes are independent (Fig. 3c,d). To follow up on the latter point, we compared damage in mtDNA with that in nDNA. In control PBMCs, DNA damage level in the two genomes correlated strongly (R = 0.86, P < 0.0001), while there was no correlation between damage in mtDNA and nDNA in HD (Fig. 3e). Given the elevated nDNA damage in HD, we investigated potential correlation between nDNA damage and clinical symptoms, represented by the TFC score. Interestingly, TFC displayed an inverse correlation with the nDNA damage level in patient PBMCs (Fig. 3f). Additionally, the duration of disease (time from onset to PBMC collection) correlated with nDNA damage (R = 0.045, P < 0.05). These results demonstrate that nDNA damage emerges as a promising novel biomarker of systemic alterations in HD.

## Discussion

In this study we have studied PBMCs from HD patients and healthy controls, and identified elevated nDNA damage and alterations in mitochondrial parameters in HD. Although mitochondrial aberrations previously have been confirmed in patient samples, this has not been established as suitable biomarkers for monitoring HD progression and effect of putative therapy. The correlation between the clinical score and DNA damage indicates that mHTT has a powerful systemic impact on DNA integrity in HD. Signs of early mitochondrial dysfunction was evident, but did not correlate with clinical score.

Associated side effects of mitochondrial dysfunction are oxidative stress and DNA damage. Urinary 8-oxoguanine is a marker of endogenous oxidative stress and was found to correlate with disease progression in the R6/2 HD model, as well as responding to therapy^31^. Impaired genome integrity has also been identified previously in caudate region of post-mortem HD brains^32^. Chen and co-workers demonstrated elevated genomic 8-oxoguanine in leukocytes from HD^33^, which is in contradiction to our data (Supplementary Fig. S1), as well as data from Borowsky *et al*.^34^. Although we don’t know the reason for the discrepancy, it is worth noting that neutrophils in general exhibit reduced genomic integrity compared to lymphocytes^35^. It may therefore be speculated that the neutrophile:lymphocyte composition vary in the two studies. Different leukocyte isolation procedures might also explain the lower mtDNA copy number in HD leukocytes found by Jędrak and co-workers^36^. Several observations herein are indicative of reduced mitochondrial activity in HD. The expression level of *SIRT1*, which supports mitochondrial biogenesis via deacetylating PGC-1α^37^, is lower in HD than in control PBMCs (Fig. 3a). This reduction is paralleled by reduced p53 levels, reduced citrate synthase and importantly, reduced level of mtDNA damage. One limitation of our study is that we did not include real-time analyses of PBMCs mitochondrial activity. However, Mejia and coworkers found strongly reduced mitochondrial activity in transformed lymphoblasts from HD patients, compared to controls. Despite this reduction the HD cells were proficient for ETC capacity, similarly as PBMCs in our study^38^.

We previously found that mitochondrial metabolism influences mtDNA integrity to the same extent as sub-lethal oxidative stress^28^. We do not believe that the epigenetic modifications contribute to the mtDNA damage signal^39^, and accordingly there was not an overall difference in epigenetic markers in genomic DNA from HD and controls (Supplementary Fig. S1). In view of these empirical data, we thus believe that the relatively spared mtDNA in HD is caused by an altered metabolic activity.

Interestingly, the strong correlation observed in control PBMCs between nDNA and mtDNA damage was abolished in HD, mainly due to strongly increased nDNA damage.

Different chromatin packing is one plausible explanation for the altered gene expression and sensitivity to nDNA damage in HD. HTT has been found to interact with and stimulate PRMT5, an arginine methyltransferase that affects chromatin packing via it’s methylation of arginine 3 of histone 2A and histone 4. This interaction was attenuated by the polyQ mutation, resulting in reduced dimethylation of H2A/H4R3 in HD patients and an HD mouse model^40^.

Reduced DNA repair activity in HD is implicated by the gene expression data (Fig. 3a) and could underlie the high nDNA damage level in HD. Emerging data infer that DNA repair factors reflect metabolism of the cell, OGG1^41^, Neil1^42^, p53^43^ are examples that respond to metabolic alterations in the cell. These alterations are interesting in view of the disproportional complex I in HD compared to controls (Supplementary Table S2).

Efficient ATP production depends on organization of ETC complexes into supercomplexes^44^, and altered stoichiometry of complex I relative to for example complex IV might have implications for intracellular ATP. The repair of nDNA was found to be relatively more dependent on intracellular ATP than repair of mtDNA^45^. Together, the indications of altered metabolism and disproportional ETC machinery are coherent with increased accumulation of nDNA damage in HD.

Earlier reports of ETC dysfunction in HD include reduction in complex III^32^, complex II, III and IV^46^, complex I^47^, as well as decreased COX activity^48^, which is in contrast to the normal ETC activities found here, beside a reduced level of SDHB (complex II) (Fig. 3b). This is indicative of a redundant capacity of ETC, where not all subunits are forming functional ETC complexes. A parallel phenomenon is illustrated by the strong increase (4-fold) in ETC activity upon cytomegalovirus infection that did not involve increased level of specific ETC subunits^49^. The ETC capacity as a good marker for mitochondrial activity is thus questionable. This is in contrast to the strong effect of ETC genes as shown by GO analyses (Supplementary Table S1), and implies that expression levels of ETC subunits rather than ETC capacity are early markers for mitochondrial dysfunction HD PBMC’s. We find it interesting that the complex II subunit SDHB is down-regulated in HD PBMCs. Systemic inhibition of complex II induces HD- like pathology^17^, and the SDHB (also known as Ip) is specifically targeted^20^. In particular, SDHB protects against 3-nitropropionic acid-induced striatal degeneration. Interestingly, SDHB is suppressed in monocytes exposed to inflammation/hypoxia in an APOBEC3-mediated manner^50^. GO analyses identified altered hypoxic signalling in the HD although not in the patients with highest CAG size (Supplementary Table S1).

The lack of correlation between SDHB and disease progression is interesting and motivates further evaluation of SDHB as a biomarker for the asymptomatic state of the disease. This is a task we are currently pursuing.

In summary, our results confirm other publications that peripheral cells are affected in HD. In addition to biomarkers found in cited reports above, mHTT protein fragments^51^ and inflammatory markers^52^ are identified in HD plasma. Importantly, we discover that nDNA damage in PBMCs correlates with duration of disease as well as TFC score. Consequently, future therapy may not necessarily need to target affected brain areas in HD. PBMCs provide an easy-accessible source that has a promising potential to be used in the future follow-up of patients, to monitor therapeutic interventions.

## Methods

### Patient material and sample collection

All work involving human samples was carried out in accordance with the World Medical Association Declaration of Helsinki and was approved by the Ethical Committee of the General University Hospital in Prague, approved project COST LD15099 (2015–2017) and Czech-Norwegian Research Programme (CZ09) 7F14308 (2014–2017). The written informed consent was obtained from patients as well as control subjects.

Transfer and analyses of human samples in Norway was approved by South-East Regional Ethical Committee (REK HSØ). Approved project no: REK 2015/1876 A.

### PBMCs

Peripheral blood was taken from healthy controls and patients with confirmed HD. Within 1-2 h after collection, whole fraction of intact PBMCs was isolated from EDTA-blood by density medium centrifugation at 25 °C, using HistopaqueR1077 Hybri-Max TM (Sigma) following a standard protocol. Briefly, 6 ml of blood was layered on top of a 3 ml of Histopaque and centrifuged at 800 × g for 30 min at 25 °C. The separated mononuclear band was carefully collected (~1 ml), resuspended in 10 ml PBS (137 mM NaCl, 2.7 mM KCl, 4.3 mM Na_2_HPO_4_.12H_2_O, 1.5 mM KH_2_PO_4_) and centrifuged again at 800 × g for 20 min. The pellet was washed twice in same conditions. Finally, the dry pellet was stored at −80 °C until use. For enzymatic and protein analyses samples from identical isolation of cells were used.

### RNA isolation, real-time qPCR and gene expression analyses

Total RNA from samples was isolated using the RNeasy Kit (Qiagen) following manufacturer’s protocols with minor modifications, and evaluated by ND-1000 spectrophotometer (Nano-drop Technologies) and Agilent 2100 Bioanalyzer with Agilent RNA 6000 nano chips. cDNA was generated from at least 50 ng RNA in 20 μl total volume using High Capacity cDNA Reverse Transcription Kit (Applied Biosystems) with the program: 10 minutes at 25 °C; 120 minutes at 37 °C; 5 minutes at 85 °C.

Quantitative real-time polymerase chain reaction (qPCR) was performed with a 7900HT Fast Real-Time PCR System (Applied Biosystems) using the Power SYBR green PCR Master mix (Applied Biosystems). qPCR was carried out with DNA/cDNA with a total volume of 10 μl containing 2 × Power SYBR Green PCR master Mix, 0.5 μM of each primer and 6 ng of mtDNA/30 ng nDNA/3 ng of cDNA. All primers were designed through Primer3 (see Supplementary Table S3 for primer sequences).

qPCR performance: 50 °C for 2 minutes, 95 °C for 10 minutes, 95 °C for 15 seconds, and 60 °C for 1 minute for 40 cycles. The ΔΔCT method was used to quantify the relative gene expression using the GAPDH gene as the internal reference gene. The baseline and threshold were adjusted for each target. RNA sequencing analyses were performed by pooling equimolar amounts of RNA from controls, and three groups of HD patients: with CAG size < 40 (low-CAG), 40–50 (mid-CAG) and >50 (high-CAG). RNA sequencing was performed by Novogene, Beijing. Gene onthology (GO) analyses on resulting Fastq data were performed by Novogene. GO with a corrected P-value of < 0.05 is significantly enriched in DEGs.

### DNA isolation, DNA integrity and mtDNA copy number

Total DNA was isolated using the DNeasy Blood and Tissue Kit (Qiagen) following manufacturer’s protocols with minor modifications. mtDNA and nDNA damage was determined using the DNA damage assay described in Wang *et al*.^30^ by qPCR, using 6 and 30 ng DNA for mtDNA and nDNA analysis, respectively. Primers for *MT-RNR1* and *NDUFA9* were used to represent mtDNA and nDNA (see Supplementary Table S3 for primer sequences). CAG sizing was performed by PCR using primers (Supplementary Table S3) with a fluorescent tag designed to flank the region of the CAG repeats. A total volume of 20 μl containing 50 ng of total DNA, 5% DMSO, 1 × GeneAmp® PCR Buffer II, 1.25 mM MgCl2, 2.5 mM dNTP, 0.5 µM forward and reverse primers and 0.075 U/µl AmpliTaq Gold® DNA polymerase and the following program was used: 94 °C for 5 minutes then for 40 cycles, 94 °C for 30 seconds, 70 °C for 30 seconds, 72 °C for 30 seconds and finally 72 °C for 3 minutes. PCR products were run on a 1.2% agarose gel, the resulting band was cut out and separated by capillary electrophoresis in a POP-7 gel at 60 °C using LIZ 600 size standard. Length of the PCR products was determined by fragment analysis on an Applied Biosystems 3130 Genetic Analyzer. Data analysis was performed by GeneMapper® software V2.6.4.

mtDNA copy number was determined by real-time qPCR quantification of the nuclear (*HBB*) and mitochondrial (*MT-RNR1*) (see Supplementary Table S3) templates.

### Western analyses

PBMC pellets (about 20 μl) were resuspended and incubated on ice in 2.5 × their volume of RIPA buffer (50 mm Tris/HCl (pH 7.4), 150 mm NaCl, 1 mm PMSF, 1 mm EDTA, 1% Triton X-100, 1% SDS (v/v) and 1% (v/v) Protease Inhibitor Cocktail (Sigma-Aldrich) for 20 minutes, vortexed every 5 minutes and then centrifuged for 20 min at 51,000 × g (4 °C). The protein concentration in supernatant was determined^53^ and proteins (20 μg per well) and a molecular weight marker (See Blue Plus2 Prestained Standard, Invitrogen) were separated by 12% SDS-PAGE^54^ (Mini-Protean System (Bio-Rad)). Protein was mixed with loading buffer (50 mm Tris/HCl (pH 6.8), 12% (v/v) glycerol, 4% SDS, 2% (v/v) 2-mercaptoethanol and 0.01% (w/v) Bromphenol Blue) for 30 minutes at 37 °C. Proteins were subsequently semidry electroblotted onto PVDF membranes (Merck), membranes air-dried overnight, rinsed with 100% methanol (v/v) and blocked in TBS (Tris-Buffered Saline) with 5% non-fat dried milk for 1 hour. Membranes were incubated with primary antibodies (see below) in TBS containing 0.1% (v/v) Tween-20 and 2% non-fat dried milk overnight at 4 °C. Secondary detection was carried out with peroxidase conjugated secondary antibodies (Sigma-Aldrich) in TBS containing 0.1% (v/v) Tween-20 and 2% non-fat dried milk for 1 hour. The proteins were then visualized with the Super Signal West Femto Maximum Sensitivity Substrate (Thermo Scientific) using Syngene Imaging System and the intensity of signal was quantified with the Quantity One 1-D Analysis Software (Bio-Rad) for further analysis.

Primary antibodies: PDH antibody cocktail MSP02/C0611 (1:2000); Complex III subunit Core 2 monoclonal antibody MS304/D1129 (1:10000); Complex II subunit 30 kDa Ip monoclonal antibody MS203/D1205 (1:2000); Complex I subunit NDUFB6 monoclonal antibody MS108 (1:2000), all Mitosciences; Anti-ATPB antibody [3D5] - Mitochondrial Marker ab14730 (1:1000); Ms mAb to MTCO2 (12C4F12) ab10258 (1:10000); Anti-Superoxide Dismutase 1 antibody [SOD1] ab20926 (1:2000); Anti-GAPDH antibody [6C5] ab8245 (1:3333); Anti-MRPS31 antibody [EPR10707] ab167406 (1:3333); Anti-Citrate synthetase antibody [2H8BB6] ab128564 (1:2500) - all Abcam; p53 Antibody 9282(1:3333), Cell Signaling. Secondary antibodies: Anti-Mouse IgG (whole molecule)-Peroxidase antibody produced in goat A8924 (1:2500); Anti-Rabbit IgG (whole molecule)-Peroxidase antibody produced in goat A0545 (1:2500), Sigma-Aldrich.

### Respiratory chain complexes and citrate synthase activity

Prior to measurements, dry pellets of isolated PBMCs were resuspended in PBS to concentration of protein approximately 3-4 mg/ml, determined as described in^55^. The specific activity of NADH-decylubiquinone oxidoreductase (NQR) (complex I), succinate decylubiquinone DCPIP reductase (SQR) (complex II) and cytochrome c oxidase (complex IV) were measured as was described previously^56,57^ using a Shimadzu 2401 UV-VIS Spectrophotometer. Briefly, to disrupt the mitochondrial membranes for complex I measurement 100 µg of the cell suspension was first lysed by 3 min incubation in distilled water. Rotenone-sensitive complex I activity was measured in assay medium with final volume of 1 ml (50 mM TRIS, pH 8.1, 2.5 mg/ml BSA, 0.3 mM KCN, 0.1 mM NADH, 50 µM decylubiquinone without and with 3 µM rotenone) and followed the decrease in absorbance at 340 nm due to the NADH oxidation (ε = 6.22 mM^−1^ cm^−1^)

Complex II activity was measured in 1 ml of assay medium containing 10 mM potassium phosphate pH 7.8, 2 mM EDTA, 1 mg/ml BSA, 200 µg of cell protein 0.3 mM KCN, 10 mM succinate, 3 µM rotenone, 0.2 mM ATP, 80uM DCPIP (2,6-dichlorophenolindophenol), 1 µM Antimycin, 50 µM decylubiquinone). The decrease in absorbance at 600 nm due to the oxidation of DCPIP at 600 nm (ε = 20.1 mM^−1^ cm^−1^) was recorded.

For determination of complex IV and CS enzyme activity, the cell suspension was solubilized by incubation with 1.5% n-dodecyl-β-D-maltoside for 15 minutes at 4 °C, then centrifuged for 10 minutes at 10,000 g and resulting supernatant used for activity assay.

Complex IV activity was measured by incubating of 100 µg of cell supernatant protein in 1 ml of assay medium (40 mM potassium phosphate, pH 7.0; 1 mg/ml BSA; 25 µM reduced cytochrome c) by following the oxidation of cytochrome c (II) at 550 nm (ε = 19.6 mM^−1^ cm^−1^). All assays were performed at 37 °C. Citrate synthase was measured with 100 µg of supernatant according to Srere^58^.

### Statistics

All statistics were carried out in GraphPad Prism. Calculation of statistical significance was done using un-paired T-test without assuming consistent standard deviation. Multiple comparisons were corrected for using the Holm Sidak method with alpha = 0.05. Correlation analysis was calculated by Pearson method at 95% confidence interval. For enzymatic activity, all dependent variables were log-transformed (log_e_). Only in the case of COX we used y = −1/x.

For western blot analysis, relationships between dependent variables were examined using linear mixed effects models with interactions. Random effect of a set of measurements (of an experiment) was also considered. Dependent variables were transformed using Box-Cox transformations. For western blot analysis, box-cox power transformation was used with the following lambdas: NDUFB6:1 (identity), SDHB: 0.25, CORE2: 0.25, COX2: 2, ATPbeta: 0.25, PDH E2:0.5, E1a: 0 (log_e_), SOD1: −0.9, p53:1 (identity), MRPS31: 0.25, CS: −0.53, GAPDH: 0 (log_e_).

### Data availability

Data that is approved for sharing to destined country is available from the corresponding author upon request.

## Electronic supplementary material

Supplementary Information
