# Supplementary material for: Increased nuclear DNA damage precedes mitochondrial dysfunction in peripheral blood mononuclear cells from Huntington’s disease patients
Source: Sci Rep. 2018 Jun 29;8:9817. doi: 10.1038/s41598-018-27985-y (PMC6026140; doi:10.1038/s41598-018-27985-y)
Supplement: Supplementary file 1 — Supplementary Information [file 41598_2018_27985_MOESM1_ESM.pdf]

Increased nuclear DNA damage precedes mitochondrial dysfunction in peripheral blood mononuclear cells from Huntington's disease patients

[1,2]Georgina Askeland, [3]Zaneta Dosoudilova, [3]Marie Rodinova, [4]Jiri Klempir, [4]Irena Liskova, [5]Anna Kuśnierczyk, [2,5]Magnar Bjørås, [2]Gaute Nesse, [2]Arne Klungland, [3]Hana Hansikova,[1]Lars Eide*

[1]Department of Medical Biochemistry, Institute of Clinical Medicine, University of Oslo; [2]Department of Microbiology, Oslo University Hospital; [3]Department of Pediatrics and Adolescent Medicine and [4]Department of Neurology and Centre of Clinical Neuroscience, First Faculty of Medicine, Charles University in Prague and General University Hospital in Prague, Czech Republic; [5]Proteomics and Metabolomics Core Facility, PROMEC, Department of Clinical and Molecular Medicine, Norwegian University of Science and Technology, Trondheim

Supplementary information

Supplementary Table S1
Supplementary Table S2
Supplementary Table S3
Supplementary Figure S1

Suppementary Table S1.
Gene onthology analyses

Description	GO accession	<40			40-50			>50		
		Rank	P-value	DEG's	Rank	P-value	DEG's	Rank	P-value	DEG's
electron transport chain	GO:0022900	529	0.012407	16	172	0.00045731	14	62	0.0055547	12
mitochondrial membrane	GO:0031966	405	0.0059333	52	485	0.025069	29	118	0.037167	27
oxidative phosphorylation	GO:0006119	1202	0.13238	10	287	0.0036619	10	92	0.017259	9
response to hypoxia	GO:0001666	525	0.012109	27	403	0.01484	16	221	0.15514	13
response to oxidative stress	GO:0006979	386	0.0046547	35	68	1.0363e-06	29	33	0.0012887	22

RNA sequencing of HD grouped according to CAG size unravels that oxidative stress response genes, mitochondrial genes and hypoxia reponse genes being affected in a CAG-dependent manner. RNA from PBMC were pooled into low-CAG (<40), mid-CAG (40-50) and high-CAG (>50) category..

Suppementary Table S2.
Correlation analyses

	CS_act	cI_act	cII_act
cIV_act	0,78**<br>0,70**	0,699**<br>n.s.	0,76**<br>0,80**
CS_act		0,66**<br>n.s.	0,88**<br>0,78**
cI_act			0,59**<br>n.s.

Activities of citrate synthase (CS_act), complex I (cI_act; NQR), complex II (cII_act; SQR), complex IV (cIV_act; COX) were subjected to Pearson correlation analyses. The table gives the resulting R values for controls (black font, n=34), and HD (grey font; n=17). **p < 0.002; n.s. : non-significant.

Supplementary Table S3.
Primer sequences

Loci	Forward	Reverse	Purpose
CYCS	CATGGCCCCTCCCATCTACA	ATCTTGAGCCCCATGCGTTT	Gene expression
D-LOOP	CACCATCCTCCGTGAAATCAA	CCCGGAGCGAGGAGAGTAG	Gene expression
DRP1	ACTGGAATTGTCACCCGGAG	CTGCTTCCACCCCATTTTCTTC	Gene expression
FEN1	CCAAAGGCCAGTCATCCCTC	GCATCAATGGCCACCTTACG	Gene expression
HBB	GAAGAGCCAAGGACAGGTAC	CAACTTCATCCACGTTCACC	Copy number
HTT	ATGAAGGCCTTCGAGTCCCTCAAGTCCTTC	CGGCGGCGGTGGCGGTTGCTGTTGCTGCTG	CAG sizing
MT-CYB	ACCCCCTAGGAATCACCTCC	GCCTAGGAGGTCTGGTGAGA	Gene expression
MT-ND2	GCCCTAGAAATAAACATGCTA	GGGCTATTCCTAGTTTTATT	Gene expression
MT-ND6	CAACCAGTAACTACTACTAA	ACTTTAATAGTGTAGGAAGC	Gene expression
MT-RNR1	AAACTGCTCGCCAGAACACT	CATGGGCTACACCTTGACCT	DNA damage/gene expression/copy number
NDUFA9	ATTCCCCTTGCCGCTTTTTG	ATGTGCATCCGCTCCACTTT	Gene expression
NDUFA9	GCAAGGGTCCCTATGAGAGAA	CAAGAACGAGGGGAAAAGTG	DNA damage
NEIL1	GCTGACCCTGAGCCAGAAGAT	CCCCAACTGGACCACTTCCT	Gene expression
NEIL2	ACCTGTGACATCCTGTCTGAGAAGT	TAATGATGTTCCCTAGCCCTGAGA	Gene expression
NEIL3	GGTCTCCACCCAGCTGTTAAAG	CACGTATCATTTTCATGAGGTGATG	Gene expression
OGG1	CGAGCCATCCTGGAAGAACAG	ACATATGGACATCCACGGGCAC	Gene expression
SIRT1	GGAGGATAGAGCCTCACATGC	TATGGACCTATCCGTGGCCT	Gene expression
SOD1	CTGTACCAGTGCAGGTCCTC	CCAAGTCTCCAACATGCCTCT	Gene expression
SOD2	GACAAACCTCAGCCCTAACG	GAAACCAAGCCAACCCCAAC	Gene expression
UCP2	CCTCTCCCAATGTTGCTCGT	GGCAAGGGAGGTCATCTGTC	Gene expression

Table shows sequences of primers used for various experiental methods as indicated in right column.

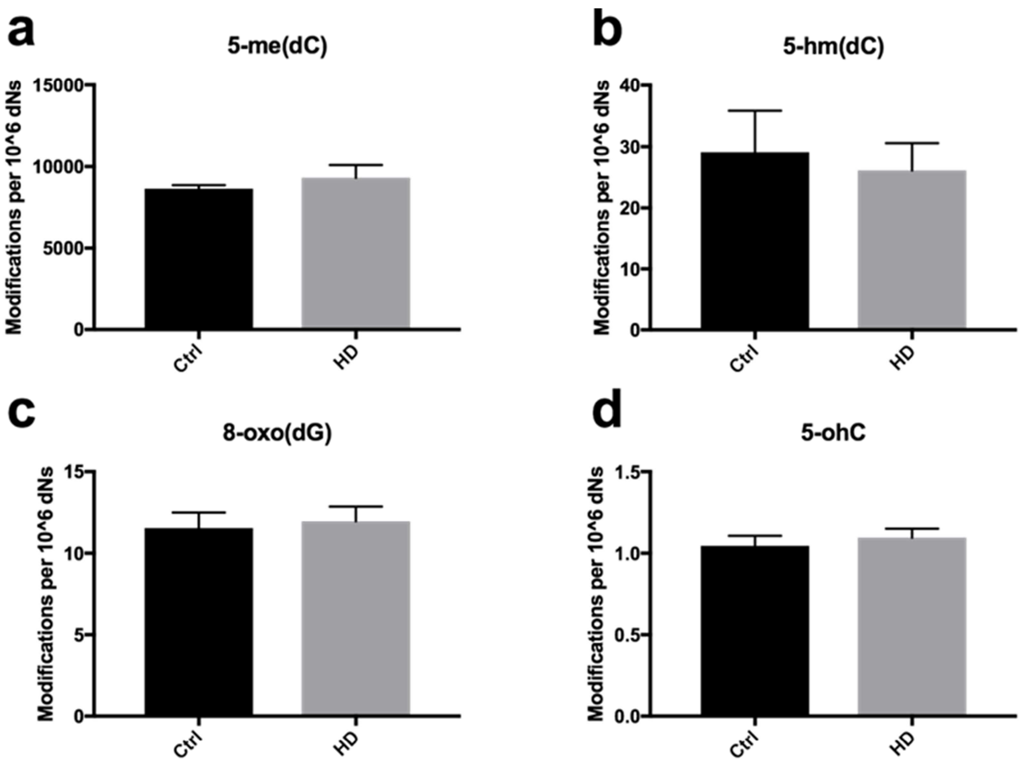

Supplementary Figure S1

Mass spectrometry analysis of DNA base modifications in Ctrl and HD lymphocytes

The levels of DNA base modifications were determined by LC-MS/MS

Frequency of the following base modifications are provided as frequency per million nt in total DNA: (a) Levels methylcytosine (5-me(dC)); (b) Levels of 5-hydroxymethylcytosine (5-hm(dC)); (c) Levels of 8-oxoguanine (8-ox Levels of 5-hydroxymethylcytosine (5-ohC).
